# Circular RNAs: Unlocking new avenues in cardiometabolic disease management

**DOI:** 10.1113/JP289839

**Published:** 2026-02-24

**Authors:** Kimberley M. Mellor, Thomas D. Donelly, Gerald L.M. Naepi, Glenn D. Wadley, Bianca C. Bernardo

**Affiliations:** ^1^ Department of Physiology University of Auckland Auckland New Zealand; ^2^ School of Exercise and Nutrition Sciences, Faculty of Health Deakin University Melbourne Victoria Australia; ^3^ Institute for Physical Activity and Nutrition, School of Exercise and Nutrition Sciences, Faculty of Health Deakin University Melbourne Victoria Australia; ^4^ Department of Cardiometabolic Health The University of Melbourne Melbourne Victoria Australia

**Keywords:** cardiometabolic disease, circRNAs, heart failure, non‐coding RNAs, therapeutic targets

## Abstract

Cardiometabolic diseases refer to a group of interrelated conditions that affect both the cardiovascular system and metabolic processes. These conditions represent a significant global health burden and often share common risk factors such as obesity, high cholesterol and insulin resistance. Cardiometabolic diseases include conditions such as hypertension, diabetes, heart failure, coronary artery disease and stroke. Recent advancements in molecular biology have highlighted the pivotal role of circular RNAs (circRNAs) in the regulation of gene expression and cellular processes. These stable, covalently closed RNA molecules act as microRNA sponges, modulating the activity of key metabolic and cardiovascular pathways. This review explores the emerging potential of circRNAs as novel therapeutic targets in cardiometabolic disease, focusing on the mechanisms by which circRNAs influence disease progression, their utility as biomarkers for early diagnosis and prognosis and the innovative therapeutic strategies being developed to harness their regulatory functions. However research into circRNAs also comes with its unique set of challenges, relating to detection, quantification, annotation variability and functional characterisation. Despite these challenges the potential for circRNAs to advance understanding and treatment of disease continues to drive research forward, paving the way for new diagnostic and therapeutic strategies to better manage cardiometabolic diseases.

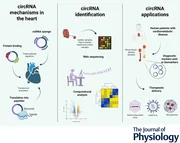

## Graphical abstract

**Abstract figure legend** In the heart circular RNAs (circRNAs) function as microRNA sponges, interact with proteins and can even undergo translation. Advances in bioinformatics now enable their identification through high‐throughput RNA sequencing, whereas computational analyses reveal differential expression in cardiac disease settings. Notably their stability and tissue‐specific expression position circRNAs as promising biomarkers for cardiometabolic diseases and as novel therapeutic targets, with delivery strategies leveraging viral vectors and nanoparticles.

## Introduction

Cardiometabolic diseases (CMD) are a spectrum of conditions affecting the heart and metabolism that primarily include cardiovascular diseases (e.g. heart failure, myocardial infarction and stroke) linked with hypertension, obesity and type 2 diabetes. Cardiovascular diseases (CVD) are the leading cause of death globally, accounting for one‐third of all deaths (Roth et al., 2020), and are a major contributor to reduced quality of life, imposing substantial burden on healthcare costs (Roth et al., 2020). Best modelling estimates from the global burden of disease (GMB) study (Chong et al., 2025) have projected a 90% increase in crude CVD prevalence over the next 25 years, with global deaths from CVD predicted to rise from 20.5 million in 2025 to 35.6 million in 2050 (Chong et al., 2025). These rises are almost entirely due to worldwide population growth and ageing (Chong et al., 2025). Furthermore high systolic blood pressure and high body mass index, due to the worldwide epidemic of obesity, remain as the two leading and modifiable risk factors for premature cardiovascular deaths (Chong et al., 2025; Koskinas et al., 2024). Thus despite nearly a century of medical progress new therapies are still needed to treat CMD.

Current therapies for CMD commonly include one or more of lipid‐lowering statins, anti‐hypertensive angiotensin‐converting enzyme (ACE) inhibitors, anti‐arrhythmia beta‐blockers and platelet aggregation inhibitors such as aspirin (Virani et al., 2023). There is also emerging adoption of anti‐diabetic medications known to have cardiovascular benefits independent of their glucose‐lowering effects, such as glucagon‐like peptide‐1 receptor agonists (Lincoff et al., 2023) and sodium‐glucose co‐transporter 2 inhibitors (Zinman et al., 2015). Not only are these medications prescribed for CMD patients with type 2 diabetes, but they are also recommended for CMD patient groups without diabetes (Lincoff et al., 2023; Virani et al., 2023). The adoption of these newer medications reflects, in part, the recognition that CMD is not confined to the cardiovascular system, and that treating the associated systemic conditions can provide cardioprotection.

Non‐coding RNAs (ncRNAs) are RNA molecules that do not encode for a protein but can regulate gene expression either directly or indirectly (Djebali et al., 2012), process other RNA molecules (Matera et al., 2007) or act as regulators for other RNA species (Wang & Chang, 2011). Of relevance for this review is that ncRNAs are well described to play regulatory roles in both physiological and pathological cardiac hypertrophy (Bernardo et al., 2015; Ooi et al., 2014; Wadley et al., 2019; Wang et al., 2020) and CVD (reviewed in Das et al., 2020; Prestes et al., 2020). Circular RNAs (circRNAs) are a class of highly stable ncRNAs that, unlike linear RNA, form a covalently closed circular molecule. CircRNAs are highly tissue‐specific, with more than 9000 candidate circRNAs detected in the hearts of each species of mouse, rat and humans (Werfel et al., 2016). They are well recognised as playing significant biological roles in the regulation of many cellular processes, including proliferation, apoptosis, angiogenesis and cell cycle progression (reviewed in Verduci et al., 2021). Their most well‐established biological function is to act as microRNA sponges (Kristensen et al., 2019), and their high stability and tissue‐specific expression make them ideal biomarkers for CMD. Indeed their aberrant expression has been well described in diabetes (Dehghanbanadaki et al., 2023; Yan et al., 2022) and CVD (Kishore et al., 2020; Tang et al., 2020), with some circRNAs having a detrimental role in pathological cardiac remodelling (Bibi et al., 2024) and others having a cardioprotective role (Wang et al., 2016; Wang, Feng et al., 2023).

This review will examine the mechanisms by which circRNAs influence CMD progression and their utility as biomarkers for early diagnosis and prognosis. Finally the potential for circRNAs to be developed as novel therapeutic targets in cardiometabolic disease will be explored, with a particular focus on cardiac remodelling and diabetic cardiomyopathy.

### Biogenesis and molecular characteristics of circRNAs

CircRNAs are generated mainly through precursor mRNA (pre‐mRNA) back‐splicing, in which a downstream 5′ splice donor joins to an upstream 3′ splice acceptor, producing a covalently closed RNA circle that competes with canonical linear splicing of the same pre‐mRNA (Greene et al., 2017; Jeck et al., 2013; Li, Yang et al., 2018). Back‐splicing is favoured when introns flanking the circularised exons contain inverted repeat elements (e.g. Alu repeats) that base‐pair to juxtapose splice sites, or when dimerising RNA‐binding proteins bridge the introns (Chen, 2020). A second pathway of circRNA generation involves exon skipping, where exon‐containing lariat intermediates, which are created as a byproduct of the normal splicing process, undergo internal splicing to yield circular products (Barrett et al., 2015). Intronic circRNAs can also arise directly from intronic lariats. The intron that is removed under usual splicing conditions escapes debranching due to conserved GU‐rich and C‐rich sequences (Zhang et al., 2013). It is trimmed and remains as a stable circular intronic RNA.

Structurally circRNAs lack free 5′ and 3′ ends and instead form covalently closed loops. This construction renders them highly resistant to exonucleases such as RNase R and generally results in longer half‐lives than their linear counterparts, allowing them to accumulate to high steady‐state levels in differentiated tissues (Jeck & Sharpless, 2014). Many circRNAs are more stable than the host mRNA and exhibit pronounced tissue‐, cell‐type‐ and developmental‐stage specificity (Greene et al., 2017).

CircRNAs are commonly classified based on their genomic origin. Exonic circRNAs, composed solely of exons, represent the majority of circRNAs recorded and are typically enriched in the cytoplasm (Ragan et al., 2019). They commonly act as microRNA (miRNA) sponges and RNA‐binding protein scaffolds (Hansen et al., 2013). Intronic circRNAs localise predominantly to the nucleus and can enhance transcription of their parental genes (Zhang et al., 2013). Exon–intron circRNAs contain both exonic and intronic sequences, interact with U1 small nuclear ribonucleoproteins and RNA polymerase II and can increase host gene expression in cis (Li et al., 2015; Zhong et al., 2024). Additional subclasses include mitochondria‐encoded circRNAs, for which emerging evidence suggests a role in the regulation of mitochondrial gene expression and function (Liu et al., 2024), and circRNAs derived from long non‐coding RNA loci (Qin et al., 2020).

### Functional roles of circRNAs in the heart

**Figure 1:**
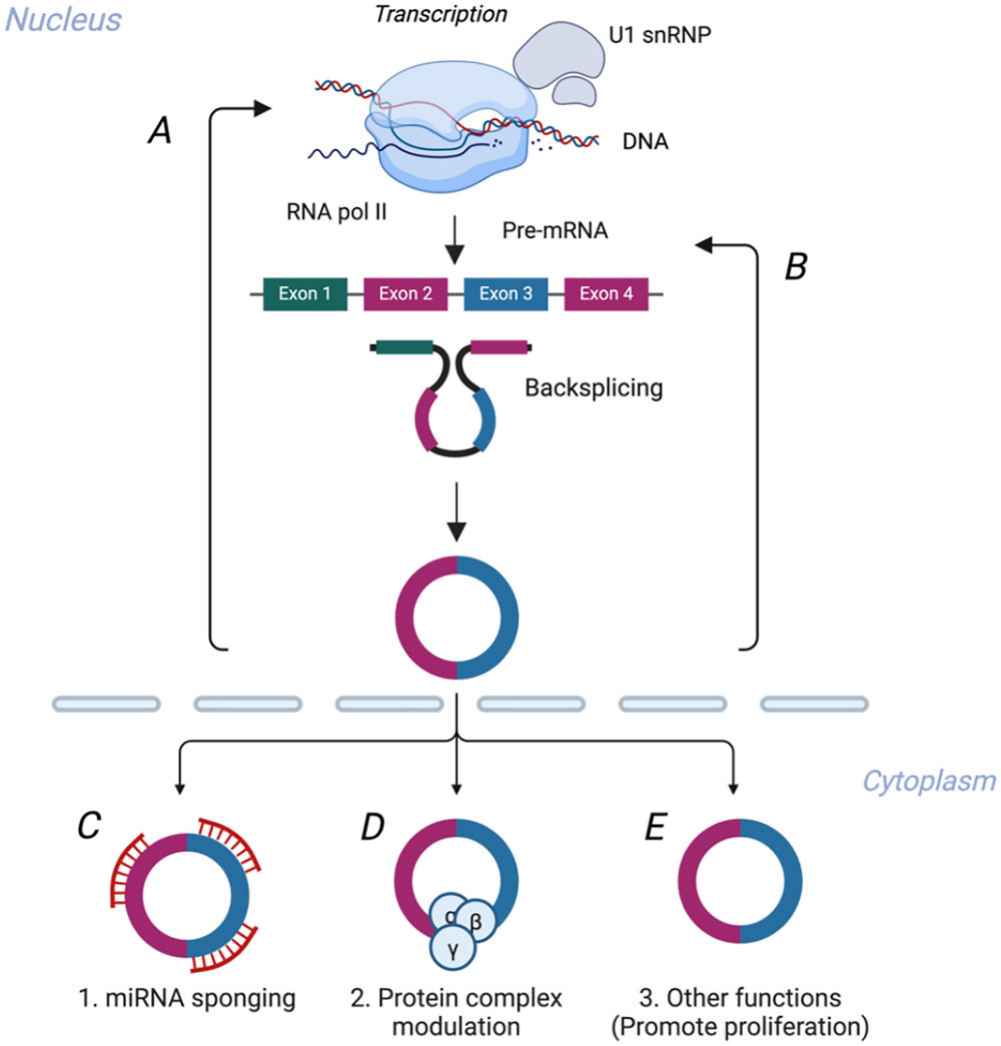
Biogenesis and functional mechanisms of circular RNAs *A*, transcriptional regulation via circRNA association with RNA Pol II and U1 snRNP. *B*, splicing modulation through competitive splicing and R‐loop formation. *C*, miRNA sponging. *D*, protein expression through RBP interaction. E, other functions including cell proliferation. Abbreviations: RNA pol II, RNA polymerase II; snRNP, U1 spliceosomal RNA; miRNA, microRNA; circRNA, circular RNA.

The functional roles of circRNAs in the heart primarily relate to gene regulation, including the regulation of transcription and splicing, miRNA sponging, protein binding and other functions, such as proliferation (Fig. 1*A* and *B*). For some circRNA roles cardiac‐specific data are not available, and extrapolation of mechanistic data from non‐cardiac settings may be informative. For example it is well established in non‐cardiac tissues that exon–intron circRNAs increase gene transcription of the transcriptional machinery (e.g. RNA polymerase II) (Li et al., 2015). The exon–intron class of circRNAs (also known as EIciRNAs) is exclusively localised in the nucleus and plays an important physiological role in fine‐tuning transcription processes (reviewed in Yin et al., 2022). Similarly studies in non‐cardiac tissues have shown that the generation of circRNAs via pre‐mRNA back‐splicing can influence the expression of the pre‐mRNA substrate through competing with linear mRNA splicing. The competition between circRNA biogenesis and host gene transcription appears particularly dominant in neural‐related genes and may play a role in neurodegenerative disorders (Ashwal‐Fluss et al., 2014). Additionally circRNAs can bind genomic DNA to form R‐loops that slow transcription and promote alternative splicing of the host gene (Ashwal‐Fluss et al., 2014). CircR‐loops are DNA–RNA hybrids initially identified in the mitochondria with influence on the rate of mtDNA synthesis (Su et al., 2024). Physiological roles for circR‐loops include maintenance of transcription and chromatin structure. However accumulation of pathological circR‐loops has been linked to DNA damage in cancer and neurological disorders (for comprehensive review see Su et al., 2024). Data on circR‐loops in cardiac settings are lacking, and significant opportunity exists in advancing understanding of the pathophysiological role of circR‐loops in cardiac disease.

The more well‐characterised role of circRNA in the heart relates to sponging of miRNAs. CircRNAs sequester miRNAs through complementary base‐pairing at miRNA response elements, thereby preventing miRNAs from binding to their target mRNAs (Fig. 1*C*). This competitive endogenous RNA mechanism requires circRNAs to contain multiple miRNA binding sites to regulate gene expression effectively (Hoffmann et al., 2023). CircRNA sponging of miRNAs in cardiac tissue has been shown to influence apoptosis, cardiomyocyte growth, mitochondrial respiration and myocardial fibrosis (Wang et al., 2016; Li, Ding et al., 2018; Hu et al., 2024), and is discussed in more detail in relation to cardiac pathophysiological settings below.

A role for circRNA in regulating protein stability, localisation and function by preventing protein degradation or facilitating nuclear translocation has been demonstrated (Fig. 1*D*) (reviewed by Ward et al., 2021). Interaction of circRNAs with RNA‐binding proteins can regulate transcriptional machinery components AGO2 and RNA Pol II, splicing‐related protein Mbl (which competes for binding with circ‐Mbl), cell cycle regulators such as CDK2 and p21 (which form a ternary complex with circFoxo3), the oncofetal tumour marker IMP3 (which associates with a circRNA subfamily involved in posttranscriptional regulation) and the RNA‐binding protein HuR (which binds to circPABPN1) (reviewed in Huang et al., 2020). Given that most of these mechanisms underlie fundamental cellular processes it is likely that these roles play out similarly in cardiac tissue. However further investigation in cardiac settings is warranted to advance our understanding of cardiac‐specific roles of circRNAs.

### CircRNA dysregulation underlying adaptive and maladaptive cardiac remodelling

CircRNAs are key regulators in heart pathophysiology, playing crucial roles in cardiac remodelling and heart failure by influencing processes such as cell death, hypertrophy and fibrosis, as well as modulating key signalling pathways. In the heart, dysregulated circRNA expression is significantly associated with various cardiac pathologies (see reviews by Bibi et al., 2024; Ward et al., 2021; You et al., 2025). The first circRNA that was found to have a functional role in the heart was heart‐related circRNA (HRCR) (Wang et al., 2016). In failing mouse hearts, HRCR gene expression was significantly decreased relative to that observed in control hearts. Mechanistically HRCR acts as a miR‐223 sponge to regulate cardiac hypertrophy and heart failure via inhibition of the protein ARC (apoptosis inhibitor with CARD domain), supporting the notion that circRNA‐driven sponging constitutes a general regulatory phenomenon. The cardiac hypertrophy‐related circRNA (circRNA CHRC) is a key regulator in the progression of heart failure, acting, in part, through its competing endogenous RNA function by interacting with the miR‐431‐5p/Kruppel‐like factor 15 axis, important for cardiac hypertrophy and fibrosis (Hu et al., 2024). Evidence indicates that modulation of circRNA sponging within cardiomyocytes could influence outcomes in myocardial infarction. Suppression of circNCX1in mouse hearts has been demonstrated to reduce cardiac ischaemia–reperfusion‐induced injury by sponging miR‐133a‐3p, which would otherwise promote cardiomyocyte apoptosis that is significant in myocardial infarction (Li, Ding et al., 2018). Within dilated cardiomyopathy it has been indicated that altered gene expression of the ryanodine receptor 2 (RYR2) gene leads to circRYR2_71 circRYR2_95 downregulation, causing altered amino acid sequence and disruption of protein structure and function (Dong et al., 2020). In the setting of cardiac senescence, circ‐Foxo3 facilitates cellular ageing processes by forming complexes with senescence‐associated proteins (Du et al., 2016). In patients with hypertrophic cardiomyopathy expression of circZFPM2 was upregulated. However somewhat surprisingly silencing circZFPM2 *in vitro* was shown to induce cardiomyocyte hypertrophy and impair mitochondrial respiratory function, resulting in elevated reactive oxygen species generation and increased apoptotic cell death (Neufeldt et al., 2024). Conversely its further overexpression rescued cardiomyocyte function in human cardiac organoids, suggesting that circZFPM2 activation in hypertrophic cardiomyopathy is compensatory and protective in nature (Neufeldt et al., 2024).

In a setting of exercise‐induced cardiac growth the circRNA circUtrn was found to be upregulated in cardiomyocytes in swim‐trained adult mice and in the serum of athletes after long‐term exercise (Wang, Feng et al., 2023). *In vivo* inhibition of circUtrn suppressed the development of cardiac hypertrophy and decreased the expression level of proliferation markers following swim‐training in mice, supporting the requirement of circUtrn for exercise‐induced physiological hypertrophic growth of cardiac tissue (Wang, Feng et al., 2023). The protective effects of circUtrn were demonstrated through its overexpression in an ischaemia/reperfusion mouse model, with improvements in cardiac function and attenuation of cardiac fibrosis (Wang, Feng et al., 2023). The mechanism by which circUtrn promotes cardiomyocyte growth and survival is through serine/threonine protein phosphatase PP5 (Wang, Feng et al., 2023). RNA‐protein interaction studies demonstrate that circUtrn directly binds to PP5 in cardiomyocytes, positioning PP5 as a key downstream effector through which circUtrn exerts its cardioprotective signalling (Wang, Feng et al., 2023). This interaction enables circUtrn to regulate PP5 protein stability through a ubiquitin‐proteasome‐dependent mechanism, influencing downstream signalling such as the mitogen‑activated protein kinase/extracellular signal‑regulated kinase (MAPK/ERK) pathway (Wang, Feng et al., 2023).

Cardiac dysfunction often coexists alongside other cardiometabolic complications such as skeletal muscle atrophy due to several related factors, including reduced physical activity, malnutrition, systemic inflammation, metabolic and hormonal derangements and oxidative stress (Philippou et al., 2020). Although there are no reports directly linking circRNAs in cardiac dysfunction to muscle atrophy, there is evidence linking skeletal muscle circRNAs in the regulation of muscle wasting. For example skeletal muscle circDdb1 expression is upregulated in rodent models of muscle atrophy (Zhu et al., 2024) and promotes muscle atrophy via activation of eukaryotic elongation factor 2 (eEF2) phosphorylation that then reduces protein translation (Zhu et al., 2024).

Collectively these findings underscore the diverse and critical roles of circRNAs in adaptive and maladaptive cardiac hypertrophy and highlight their potential as diagnostic biomarkers and therapeutic targets.

### Therapeutic potential of circRNAs in diabetic cardiomyopathy

Recent studies have reported a role for circRNAs in mediating various aspects of diabetic cardiomyopathy. Therapeutic potential has been demonstrated for several targets, primarily via genetic manipulation of cardiac‐expressed circRNAs. For example the potential for therapeutic targeting of circHIPK3 in diabetic cardiomyopathy was demonstrated by treating streptozotocin‐induced diabetic mice with adeno‐associated virus serotype 9 (AAV9) delivery of shRNA to knock down circHIPK3 in the heart. Cardiac fibrosis, myocardial remodelling and systolic dysfunction were rescued by circHIPK3 knockdown in diabetic mice (Wang et al., 2021). Several studies have shown that circRNAs are involved in cardiomyocyte cell death in diabetes (Shao et al., 2022; Shen et al., 2024). *In vitro* mechanistic experiments have mapped the molecular signalling of circMAP3K5 in mediating high glucose‐induced cardiomyocyte cell death (Shen et al., 2024). CircMAP3K5 targets miR‐22‐3p, thus relieving its inhibitory effect on death‐associated protein kinase 2 (DAPK2). Knockdown of circMAP3K5 via siRNA fully attenuated high glucose‐induced apoptotic markers Bax:Bcl2 ratio and cleaved caspase‐3 (Shen et al., 2024), suggesting that the inhibition of circMAP3K5 could be a viable therapeutic target. Further *in vivo* experiments are now warranted. Another circRNA, CDR1as, has similarly been shown to regulate cardiomyocyte death in diabetes. CDR1as is upregulated in streptozotocin‐induced diabetic rat hearts, and siRNA knockdown of CDR1as attenuated high glucose‐induced cardiomyocyte cell death *in vitro* via the hippo signalling pathway (Shao et al., 2022). *In vivo*, AAV9 delivery of shRNA targeting CDR1as achieved myocardial knockdown of CDR1as and rescued systolic dysfunction in diabetic mice (Shao et al., 2022). A role for the circRNA, DICAR, in mediating pyroptosis (i.e. a highly inflammatory form of programmed cell death) in diabetic cardiomyopathy has been demonstrated using gain‐ and loss‐of‐function DICAR genetic mouse models (Yuan et al., 2023). Further, a synthetised DICAR fragment was shown to attenuate cardiomyocyte pyroptosis *in vitro* – a promising first step in translation of targeting circRNAs by non‐genetic means (Yuan et al., 2023). Emerging evidence suggests that circRNAs are also involved in PANoptosis, a form of programmed cell death involving multicomplexes called panoptosomes. Downregulation of circOGDH via AAV9‐siRNA delivery attenuated PANapoptosis and partially restored cardiac function in diabetic mice. Conversely upregulation of circOGDH via lentiviral delivery exacerbated diabetic cardiomyopathy (Guan et al., 2025). *In vivo* gain‐ and loss‐of‐function AAV9 experiments similarly demonstrated involvement of circ_0 05007 in myocardial fibrosis, cardiac hypertrophy, ferroptosis and cardiac dysfunction in diabetic mice (Ni et al., 2024). To date targeting cardiac circRNAs has primarily used a viral gene delivery approach in experimental settings. Translation of circRNA targeting for cardiac disease will most likely employ alternative delivery systems such as lipid nanoparticles (LNPs) or extracellular vesicles (EVs) due to the challenges of viral gene delivery in humans (discussed further in the next section and see reviews (Bass‐Stringer et al., 2018; Sasaki et al., 2023; Weeks & Bernardo, 2025)). Specifically we have previously shown that employing AAV‐mediated gene therapy in the context of diabetic cardiomyopathy requires careful consideration of multiple factors, including the disease model, AAV serotype, vector dose and delivery route (Weeks & Bernardo, 2025). Although AAV9 has demonstrated success in high‐fat, high‐sugar diet models (Mellor et al., 2025), the application of AAV6 in streptozotocin‐induced diabetic model of heart failure has proven more challenging (Weeks et al., 2024). It was speculated that these discrepancies may reflect differences in AAV receptor availability or the role of insulin in facilitating viral uptake, highlighting the need for tailored strategies when translating AAV‐based therapies across distinct diabetic cardiomyopathy models. These considerations will also apply to circRNA–AAV‐based therapies.

Identification of circRNAs that elicit cardioprotection may hold the most promise for ease of delivery of synthetic circRNA constructs, rather than RNAi approaches. The first synthetic circRNA therapeutic has entered phase I trials and awaits safety outcomes (NCT06714253) (Zhao et al., 2025). Additionally emerging evidence suggests that circRNAs may mediate the cardioprotective effects of some common therapies (e.g. circRNA‐RBCK1 involvement in statin mechanism of action in HFpEF (Li et al., 2024)); thus optimisation of existing therapies with adjunct circRNA input may provide additional opportunities for therapeutic refinement.

### Delivery strategies for circRNA therapeutics

The most commonly used delivery platforms for circular RNAs include EVs, LNPs and AAV vectors, each offering distinct advantages for stability, targeting and translational efficiency for cardiac gene and RNA delivery (Bader et al., 2024; Bass‐Stringer et al., 2018; Gil‐Cabrerizo et al., 2024; Makkar, 2025). EVs are naturally derived carriers that encapsulate circRNAs within a lipid bilayer, conferring high biocompatibility, low immunogenicity and the potential for intrinsic or engineered tissue tropism (Ilahibaks et al., 2024). They can exhibit robust cardiac tropism for those derived from cardiac progenitor cells (Nawaz et al., 2025). In a preclinical study, EV‐mediated delivery of circWhsc1 induced cardiomyocyte proliferation and heart regeneration following myocardial infarction (Wei et al., 2023). Despite their superior targeting and demonstrated functional repair in a preclinical cardiac injury models (Nawaz et al., 2025; Wei et al., 2023), EVs remained constrained by heterogenous composition, limited scalability and natural capsid‐size restrictions that limit cargo loading (Miao & Huang, 2024). LNPs are synthetic delivery systems that efficiently protect circRNAs from degradation and facilitate cellular uptake (Swingle et al., 2025), offering advantages in scalability, manufacturing control and transient, non‐integrating expression. However systemically delivered LNPs predominantly accumulate in the liver, which reduces effective delivery to the heart and results in greater off‐target transcriptomic effects and immune activation (Bader et al., 2024). Recent optimisation of circRNA purification and LNP delivery strategies, including the development of novel ionizable lipids and lyophilisation approaches, has improved stability and *in vivo* expression, advancing the practical application of circRNA therapeutics (Broset et al., 2025).

AAV vectors have known *in vivo* transduction efficiency, especially through cardiotropic serotypes such as AAV9, enabling durable gene expression and strong functional benefits in animal models of cardiac disease (Mellor et al., 2025; Wang, Feng et al., 2023). Yet AAVs face significant challenges, including preexisting neutralising antibodies, dose‐dependent toxicity and a strict ∼4.7 kb packaging limit that restricts therapeutic payload size (Bass‐Stringer et al., 2018; Byrne et al., 2025). Collectively these platforms present complementary trade‑offs between safety, expression longevity, cargo capacity and translational scalability, underscoring the importance of platform selection based on therapeutic context.

### CircRNAs as biomarkers in cardiometabolic diseases

Circular RNAs possess several characteristics that make them promising candidates as biomarkers of disease. CircRNAs are abundant in the circulation and have a strong stability profile as their closed circular structure provides resistance to degradation by exonucleases (Nozima et al., 2025; Verduci et al., 2021). Evidence of circRNA involvement in cardiac disease mechanisms (as detailed above) strengthens the case for use as a biological indicator of disease. To date investigation into circRNAs as cardiovascular disease biomarkers has primarily focused on detecting myocardial injury in ischaemic settings (myocardial infarction, ischaemic heart disease, coronary artery disease (Nozima et al., 2025). Studies investigating circRNAs as biomarkers of heart failure are more limited.

From the few reports available several circRNAs have emerged as potential biomarkers of heart failure, although sample sizes are small and further validation is required. In a microarray plasma screen from three patients with chronic heart failure it was identified that 477 circRNAs were upregulated, and 219 circRNAs were downregulated relative to three healthy controls (Sun et al., 2020). The five leading upregulated circRNA candidates were investigated further in a cohort of 30 heart failure patients and clinical utility of two circRNAs (hsa_circ_0062960 and hsa_circ_0112085), as biomarkers of heart failure were validated with receiver operator characteristic curves AUC >0.8. A significant correlation of serum B‐type natriuretic peptide (BNP) with the circRNA hsa_circ_0062960 was reported (Sun et al., 2020). In a similar study of blood samples from five heart failure patients, 29 upregulated and 27 downregulated circRNAs were identified (relative to four healthy controls) (Han et al., 2020). Of the six circRNAs selected for further analysis using quantitative PCR (qPCR) in a cohort of 40 heart failure patients, hsa_circ_0097435 was identified as a leading candidate with the greatest extent of upregulation (∼5‐fold higher *vs*. controls) (Han et al., 2020). Although a mechanistic link between hsa_circ_0097435 and cardiomyocyte apoptosis and miRNA targeting was reported, analyses of biomarker sensitivity or specificity and correlations with heart failure severity were not performed. Substantial opportunity exists for development of circRNA biomarkers for heart failure, with initial leads identified from the studies described above and further candidates accessible in publicly available databases (e.g. exoRBase (Lai et al., 2022); circBase, circBank, circPac (Foroutan et al., 2025)). To date no studies are available that directly assess circRNA biomarker utility in diagnostic and/or prognostic prediction in cardiometabolic settings.

### Challenges and future directions

A persistent barrier to circRNA research is the lack of a unified naming convention across databases and studies, that is, including/not including gene information or project‐related nomenclature, which complicates meta‐analysis and replication (Hiam et al., 2025; Vromman et al., 2020). Recent proposals argue for a common, gene‐linked naming scheme (using a ‘circ’ prefix, explicit exon/intron details and a single reference transcript) (Tao et al., 2025). Additionally when using back‐splice‐junction (BSJ)‐based methods, different circRNAs can be indistinguishable when arising from the same linear RNA molecule (Hiam et al., 2025). Long‐read RNA sequencing helps by capturing internal sequences, improving isoform annotation and sensitivity at low abundance (Rahimi et al., 2021; Zheng et al., 2019). However current long‐read platforms trade throughput for read length and can show higher error rates, so pairing long‐ and short‐read data with orthogonal validation (RT‐PCR, Northern blotting, RNase R treatment) is recommended (Lin et al., 2025; Vromman et al., 2021; Wang, Xu et al., 2023).

Intervention studies show both overexpression and knockdown can modify various disease traits *in vivo* (He et al., 2021). Viral vectors (adenovirus, lentivirus and AAV), extracellular vesicles and nanoparticles are circRNA delivery routes (Chen et al., 2025). Although despite their stability, therapeutic circRNAs face hurdles in delivery, biodistribution, durability and safety. These issues include (i) targeted delivery with clinically scalable vectors while minimising off‐tissue exposure, (ii) innate immune responses, (iii) manufacturing and quality control (avoiding nicked/concatemeric species), (iv) dose control (i.e. frequency, toxicology) and (v) regulatory guidelines that are clear and distinct from linear RNA drugs (Chen et al., 2025; Dong et al., 2023; Hiam et al., 2025; Wang, Xu et al., 2023). Preclinical oncology and infectious‐disease models show feasibility, but rigour in safety and pharmacology is required before wider cardiometabolic use on their own (Chen et al., 2025; Conn et al., 2024). Although rather than acting as standalone agents, circRNA therapeutics show potential as adjuncts to complement current standards of care and advance cardiometabolic treatment in the future. Recent evidence demonstrates that the mechanisms of some existing drugs are intrinsically linked to circRNA modulation. For example statins improve cardiac endothelial function by upregulating circRNA‐RBCK1 (Li et al., 2024), whereas sodium‐glucose cotransporter 2 inhibitors exert protective effects through pathways involving other circRNAs (mostly through miRNA sponging) (Jarosz‐Popek et al., 2024). Utilising these interactions could enhance efficacy, reduce the required dosage of existing drugs and mitigate off‐target side effects, ultimately leading to improved clinical outcomes for patients with complex cardiometabolic conditions. Overcoming immunogenicity, off‐target effects and delivery issues need to be addressed and trialled in humans to realise this potential (Wang, Xu et al., 2023).

### Conclusion

In conclusion, CMDs are a spectrum of conditions affecting the heart and metabolism that are a major contributor to CVD, which accounts for one‐third of all deaths globally. Furthermore deaths from CVD are predicted to rise by approximately 75% over the next 25 years, primarily due to worldwide population growth and ageing. Therefore a greater understanding of the underlying molecular mechanisms and the development of newer medication options for the treatment of CMD are needed – approaches that are not only confined to the cardiovascular system but also treat metabolic disorders such as type 2 diabetes and obesity. CircRNAs are a relatively novel class of highly stable ncRNAs that are relatively tissue specific and with biological roles in the regulation of many cellular processes. Their aberrant expression has also been linked to many pathological conditions and for all these reasons they are of particular interest as biomarkers of disease and as potential therapeutics.

The high stability and abundance of circRNAs in the circulation have made them promising candidates as biomarkers of cardiovascular disease. Much of this research has so far focused on detecting myocardial injury in response to ischaemia, with some circRNA candidates identified as potential biomarkers of heart failure. However no studies have been published yet that have established the utility of circRNA as biomarkers in cardiometabolic settings.

The potential for circRNAs as therapies for CMD is probably best illustrated from their role(s) in diabetic cardiomyopathy. Preclinical studies have so far mostly utilised mouse models to manipulate expression of cardiac circRNAs. The knockdown or inhibition of aberrant circRNAs in mouse models of diabetic cardiomyopathy across several different studies has rescued myocardial dysfunction, remodelling and development of fibrosis (Ni et al., 2024; Wang et al., 2021), as well as cardiomyocyte cell death and apoptosis (Guan et al., 2025; Shao et al., 2022; Shen et al., 2024; Yuan et al., 2023). However key challenges remain to translate a promising preclinical therapy into a human study, particularly nucleic acid delivery, biodistribution, durability and safety. For these reasons the outcomes of the first synthetic circRNA therapeutic to recently enter phase I trials for safety evaluation are much anticipated. Despite these challenges circRNAs provide new avenues to better understand the development of disease along with diagnostic and therapeutic strategies to better manage and treat cardiometabolic diseases.

## Additional information

### Competing interests

The authors declare no competing financial interests.

### Author contributions

T.D.D. and G.L.M.N.: investigation, writing – original draft. K.M.M. and G.D.W.: investigation, writing – original draft, writing – review & editing. B.C.B.: conceptualisation, investigation, writing – original draft, writing – review & editing. All authors approved the final version of manuscript.

### Funding

None.

## Supporting information


Peer Review History
