# Supplementary material for: Circular RNAs: Unlocking new avenues in cardiometabolic disease management
Source: J Physiol. 2026 Feb 24;604(17):7047–58. doi: 10.1113/JP289839 (PMC13532961; doi:10.1113/JP289839)
Supplement: Supplementary file 1 [file TJP-604-7047-s001.pdf]

The Journal of Physiology

Circular RNAs: Unlocking New Avenues in Cardiometabolic Disease Management

Kimberley M Mellor, Thomas D. Donelly, Gerald L.M. Naepi, Glenn D Wadley, and Bianca C. Bernardo
DOI: 10.1113/JP289839

Corresponding author(s): Bianca Bernardo (bianca.bernardo@deakin.edu.au)

The referees have opted to remain anonymous.

Review Timeline:

Submission Date:	14-Dec-2025
Editorial Decision:	16-Jan-2026
Revision Received:	28-Jan-2026
Accepted:	03-Feb-2026

Senior Editor: Laura Bennet

Reviewing Editor: Kyle McCommis

Transaction Report:

(Note: With the exception of the correction of typographical or spelling errors that could be a source of ambiguity, letters and reports are not edited. Depending on transfer agreements, referee reports obtained elsewhere may or may not be included in this compilation. Referee reports are anonymous unless the Referee chooses to sign their reports.)

Dear Dr Bernardo,

Re: JP-SR-2025-289839 "Circular RNAs: Unlocking New Avenues in Cardiometabolic Disease Management" by Kimberley M Mellor, Thomas D. Donelly, Gerald L.M. Naepi, Glenn D Wadley, and Bianca C. Bernardo

Thank you for submitting your manuscript to The Journal of Physiology. It has been assessed by a Reviewing Editor and by 2 expert referees and we are pleased to tell you that it is acceptable for publication following satisfactory revision.

Please advise your co-authors of this decision as soon as possible.

The referee reports are copied at the end of this email.

Please address all the points raised and incorporate all requested revisions or explain in your Response to Referees why a change has not been made. We hope you will find the comments helpful and that you will be able to return your revised manuscript within 4 weeks. If you require longer than this, please contact journal staff: jp@physoc.org.

Your revised manuscript should be submitted online using the link in your Author Tasks: Link Not Available. This link is accessible via your account as Corresponding Author; it is not available to your co-authors. If this presents a problem, please contact journal staff (jp@physoc.org). Image files from the previous version are retained on the system. Please ensure you replace or remove any files that are being revised.

If you do not wish to submit a revised version of your manuscript, you must inform our journal staff (jp@physoc.org) or reply to this email to request withdrawal. Please note that a manuscript must be formally withdrawn from the peer review process at one journal before it may be submitted to another journal.

TRANSPARENT PEER REVIEW POLICY: To improve the transparency of its peer review process, The Journal of Physiology publishes online as supporting information the peer review history of all articles accepted for publication. Readers will have access to decision letters, including Editors' comments and referee reports, for each version of the manuscript, as well as any author responses to peer review comments. Referees can decide whether or not they wish to be named on the peer review history document.

ABSTRACT FIGURES: Authors may use The Journal's premium BioRender account to create/redraw their Abstract Figures (and any other suitable schematic figures). Information on how to access this account is here: https://physoc.onlinelibrary.wiley.com/journal/14697793/biorender-access.

This will enable Authors to create and download high-resolution figures. If authors have used the free BioRender service, they can use the instructions provided in the link above to download a high-resolution version suitable for publication.

The link provided should only be used for the purposes of this submission. Authors will be charged for figures created on this account if they are not related to this manuscript submission.

LANGUAGE EDITING AND SUPPORT FOR PUBLICATION: If you would like help with English language editing, or other article preparation support, Wiley Editing Services offers expert help, including English Language Editing, as well as translation, manuscript formatting, and figure formatting at www.wileyauthors.com/eeo/preparation. You can also find resources for Preparing Your Article for general guidance about writing and preparing your manuscript at www.wileyauthors.com/eeo/prepresources.

REVISION CHECKLIST: Upload a full Response to Referees file. To create your 'Response to Referees' copy all the reports, including any comments from the Senior and Reviewing Editors, into a Microsoft Word, or similar, file and respond to each point, using font or background colour to distinguish comments and responses and upload as the required file type.

Please upload two versions of your manuscript text: one with all relevant changes highlighted and one clean version with no changes tracked. The manuscript file should include all tables and figure legends, but each figure/graph should be uploaded as separate, high-resolution files.

You may also upload:

- 'Potential Cover Art' for consideration as the issue's cover image.
- Appropriate Supporting Information (Video, audio or data set: see https://jp.msubmit.net/cgi-bin/main.plex?form_type=display_requirements#supp).

We look forward to receiving your revised submission.

If you have any queries, please reply to this email and we will be pleased to advise.

Yours sincerely,

Laura Bennet
Senior Editor
The Journal of Physiology

REQUIRED ITEMS

- Please upload separate high quality figure files via the submission form.

EDITOR COMMENTS

Reviewing Editor:

Comments to the Author:
Thanks you for submitting your symposia review titled "Circular RNAs: Unlocking New Avenues in Cardiometabolic Disease Management" to The Journal of Physiology. It has been evaluated by an academic review editor and 2 external reviewers. All reviewers find the article to be interesting and likely to have influence on the field. The reviewers suggest some minor additions to briefly increase description of circRNAs in non-cardiac tissues, particularly skeletal muscle, which would help broaden the scope of the review.

REFEREE COMMENTS

Referee #1:

This is a comprehensive and timely review that effectively summarizes current knowledge of circular RNAs in cardiometabolic disease, with particular focus on their roles in cardiac remodeling and diabetic cardiomyopathy. The manuscript is well-structured, clearly written, and provides valuable insights into circRNA biogenesis, functional mechanisms, and therapeutic potential. The integration of biomarker applications and discussion of translational challenges adds practical value. The manuscript is suitable for publication in The Journal of Physiology with minor revisions.

Minor Revisions Required

1. Given the cardiometabolic disease focus and the emerging evidence linking cardiac and skeletal muscle pathophysiology in metabolic disorders, the review would benefit from briefly discussing circRNA roles in skeletal muscle atrophy. Recent evidence demonstrates that circRNAs regulate skeletal muscle function, which is particularly relevant to cardiometabolic disease where muscle wasting often co-exists with cardiac dysfunction. The authors should consider adding a brief subsection or paragraph discussing circRNA involvement in skeletal muscle atrophy, referencing recent work demonstrating that EIF4a3-induced circDdb1 promotes muscle atrophy through specific molecular mechanisms (PMID: 39412095). This addition would strengthen the manuscript's comprehensive coverage of cardiometabolic complications and provide broader physiological context for circRNA functions.

2. While the authors acknowledge AAV delivery challenges in diabetic cardiomyopathy models, the discussion of alternative delivery systems remains superficial. Given that lipid nanoparticles and extracellular vesicles are mentioned as alternatives to viral vectors, the authors should provide more specific details on their advantages, limitations, and current development status for cardiac applications. Brief mention of tissue-specific targeting strategies would be valuable.

3. Figure 1 is clear and informative; ensure all abbreviations are defined in the figure legend.

Referee #2:

This is an article prepared from the proceedings of the Australian Physiological Society meeting held in Sydney, November 2025. This mini review is a nice summary of the role of circular RNAs in transcription, splicing modulation, miRNA sponging and protein expression in non-cardiac tissue and in cardiac pathology. The review discusses the role of circular RNAs as potential therapeutic targets and biomarkers in the management of cardiometabolic diseases.

I have some minor queries or comments

1. First para. Line 84...you could add the contributions of a worldwide epidemic of obesity, particularly as a segway to the next section.

2. Line 160. What tissues/physiological processes are examples of circRNAs and R loops in non cardiac settings? You could expand on what is known about the role of circRNAs on gene expression in other systems.

3. Lines 162-3 Why are there no reports in cardiac settings ...is it unknown or not involved?

4. Line 230 please expand on how PP5 alters circUtrn function?

5. Line 257 explain "pyroptosis"?

END OF COMMENTS

EDITOR COMMENTS

Reviewing Editor:

Comments to the Author:
Thanks you for submitting your symposia review titled "Circular RNAs: Unlocking New Avenues in Cardiometabolic Disease Management" to The Journal of Physiology. It has been evaluated by an academic review editor and 2 external reviewers. All reviewers find the article to be interesting and likely to have influence on the field. The reviewers suggest some minor additions to briefly increase description of circRNAs in non-cardiac tissues, particularly skeletal muscle, which would help broaden the scope of the review.

We thank the reviewers for their thoughtful and constructive suggestions. We have carefully addressed each point, and their insights have significantly strengthened our manuscript.

REFEREE COMMENTS

Referee #1:

This is a comprehensive and timely review that effectively summarizes current knowledge of circular RNAs in cardiometabolic disease, with particular focus on their roles in cardiac remodeling and diabetic cardiomyopathy. The manuscript is well-structured, clearly written, and provides valuable insights into circRNA biogenesis, functional mechanisms, and therapeutic potential. The integration of biomarker applications and discussion of translational challenges adds practical value. The manuscript is suitable for publication in The Journal of Physiology with minor revisions.

Minor Revisions Required

1. Given the cardiometabolic disease focus and the emerging evidence linking cardiac and skeletal muscle pathophysiology in metabolic disorders, the review would benefit from briefly discussing circRNA roles in skeletal muscle atrophy. Recent evidence demonstrates that circRNAs regulate skeletal muscle function, which is particularly relevant to cardiometabolic disease where muscle wasting often co-exists with cardiac dysfunction. The authors should consider adding a brief subsection or paragraph discussing circRNA involvement in skeletal muscle atrophy, referencing recent work demonstrating that EIF4a3-induced circDdb1 promotes muscle atrophy through specific molecular mechanisms (PMID: 39412095). This addition would strengthen the manuscript's comprehensive coverage of cardiometabolic complications and provide broader physiological context for circRNA functions.

We thank the reviewer for this suggestion. We have discussed the evidence linking skeletal muscle circRNAs in the regulation of muscle wasting with the following additional paragraph:

Page 11 para 2: Cardiac dysfunction often co-exists alongside other cardiometabolic complications such as skeletal muscle atrophy that are due to several related factors including reduced physical activity, malnutrition, systemic inflammation, metabolic and hormonal derangements and oxidative stress {Philippou, 2020 #92}. Although there are no

reports directly linking circRNAs in cardiac dysfunction to muscle atrophy, there is evidence linking skeletal muscle circRNAs in the regulation of muscle wasting. For example, skeletal muscle circDdb1 expression is upregulated in rodent models of muscle atrophy {Zhu, 2024 #93} and promotes muscle atrophy via activation of eukaryotic elongation factor 2 (eEF2) phosphorylation that then reduces protein translation {Zhu, 2024 #93}.

2. While the authors acknowledge AAV delivery challenges in diabetic cardiomyopathy models, the discussion of alternative delivery systems remains superficial. Given that lipid nanoparticles and extracellular vesicles are mentioned as alternatives to viral vectors, the authors should provide more specific details on their advantages, limitations, and current development status for cardiac applications. Brief mention of tissue-specific targeting strategies would be valuable.

We now include additional commentary discussing delivery strategies for circRNA therapeutics, providing details about their limitations and advantages.

Page 13 para 3: new section on 'Delivery strategies for circRNA therapeutics'.

3. Figure 1 is clear and informative; ensure all abbreviations are defined in the figure legend.
Apologies for this oversight, we have now defined the abbreviations in the figure legend.

Referee #2:

This is an article prepared from the proceedings of the Australian Physiological Society meeting held in Sydney, November 2025. This mini review is a nice summary of the role of circular RNAs in transcription, splicing modulation, miRNA sponging and protein expression in non-cardiac tissue and in cardiac pathology. The review discusses the role of circular RNAs as potential therapeutic targets and biomarkers in the management of cardiometabolic diseases.

I have some minor queries or comments

1. First para. Line 84...you could add the contributions of a worldwide epidemic of obesity, particularly as a segway to the next section.
Thank you, we have now added this point into the Introduction.

Page 5 para 1: Furthermore, high systolic blood pressure, and high body mass index due to the worldwide epidemic of obesity, remain as the two leading and modifiable risk factors for premature cardiovascular deaths.

2. Line 160. What tissues/physiological processes are examples of circRNAs and R loops in non cardiac settings? You could expand on what is known about the role of circRNAs on gene expression in other systems.

Further discussion of the roles of circRNAs and circR-loops has been provided in revision with additional detail on specific regulatory actions evidenced in non-cardiac settings. To maintain the novel cardiometabolic focus of this mini-review we have intentionally

maintained brevity in discussion of non-cardiac aspects but now direct readers to comprehensive reviews for more detailed commentary.

Page 7 para 2 – page 8 para 1: new text in section 'Functional roles of circRNAs in the heart'

3. Lines 162-3 Why are there no reports in cardiac settings ...is it unknown or not involved?

We have now clarified this point.

Page 8 para 1: Data on circR-loops in cardiac settings are lacking and significant opportunity exists in advancing understanding of the pathophysiological role of circR-loops in cardiac disease.

4. Line 230 please expand on how PP5 alters circUtrn function?

Further discussion on the mechanism and interaction between PP5 and circUtrn has been included.

Page 11 para 1: RNA-protein interaction studies demonstrate that circUtrn directly binds to PP5 in cardiomyocytes, positioning PP5 as a key downstream effector through which circUtrn exerts its cardioprotective signalling (Wang et al., 2023a). This interaction enables circUtrn to regulate PP5 protein stability through a ubiquitin-proteasome-dependent mechanism, influencing downstream signalling such as the mitogen-activated protein kinase/extracellular signal-regulated kinase (MAPK/ERK) pathway (Wang et al., 2023a).

5. Line 257 explain "pyroptosis"?

We have now defined pyrootosis as a "a highly inflammatory form of programmed cell death" at first instance for clarity (page 12).

Dear Dr Bernardo,

Re: JP-SR-2026-289839R1 "Circular RNAs: Unlocking New Avenues in Cardiometabolic Disease Management" by
Kimberley M Mellor
Thomas D. Donelly
Gerald L.M. Naepi
Glenn D Wadley
Bianca C. Bernardo

I am pleased to tell you that your Symposium Review article has been accepted for publication in The Journal of Physiology, subject to any modifications to the text that may be required by the Journal Office to conform to House rules.

NEW POLICY: In order to improve the transparency of its peer review process, The Journal of Physiology publishes online as supporting information the peer review history of all articles accepted for publication. Readers will have access to decision letters, including all Editors' comments and referee reports, for each version of the manuscript and any author responses to peer review comments. Referees can decide whether or not they wish to be named on the peer review history document.

The last Word version of the paper submitted will be used by the Production Editors to prepare your proof. When this is ready, you will receive an email containing a link to Wiley's Online Proofing System. The proof should be checked and corrected as quickly as possible.

All queries at proof stage should be sent to tjp@wiley.com.

The accepted version of the manuscript is the version that will be published online until the copy edited and typeset version is available. Authors should note that it is too late at this point to offer corrections prior to proofing. Major corrections at proof stage, such as changes to figures, will be referred to the Reviewing Editor for approval before they can be incorporated. Only minor changes, such as to style and consistency, should be made a proof stage. Changes that need to be made after proof stage will usually require a formal correction notice.

Are you on Twitter? Once your paper is online, why not share your achievement with your followers. Please tag The Journal (@jphysiol) in any tweets and we will share your accepted paper with our 30,000+ followers!

If you would like to receive our 'Research Roundup', a monthly newsletter highlighting the cutting-edge research published in The Physiological Society's family of journals (The Journal of Physiology, Experimental Physiology and Physiological Reports), please click this link, fill in your name and email address and select 'Research Roundup':
https://www.physoc.org/journals-and-media/membernews/

Yours sincerely,

Laura Bennet
Senior Editor
The Journal of Physiology

EDITOR COMMENTS

Reviewing Editor:

Comments to the Author:
Thank you for submitting your revised review article titled "Circular RNAs: Unlocking New Avenues in Cardiometabolic Disease Management" to The Journal of Physiology. The reviewers both agree that the revisions made strengthen the review and that the article should have high impact on the field.

REFEREE COMMENTS

Referee #1:

I think all concerns have been solved. Nice Review!

Referee #2:

The authors have addressed all of my concerns. I have no further comments.

----

* IMPORTANT NOTICE ABOUT OPEN ACCESS *

To assist authors whose funding agencies mandate immediate public access to published research findings, The Journal of Physiology allows authors to pay an Open Access (OA) fee to have their papers made freely available immediately on publication.

You will receive an email from Wiley with details on how to register or log-in to Wiley Authors Services where you will be able to place an OnlineOpen order.

You can check if you funder or institution has a Wiley Open Access Account here: https://authorservices.wiley.com/author-resources/Journal-Authors/licensing-and-open-access/open-access/author-compliance-tool.html.

Your article will be made Open Access upon publication, or as soon as payment is received.
